# Herpes simplex virus 1 associated with impaired cognitive performance in adolescent patients with psychosis

**DOI:** 10.1192/j.eurpsy.2026.10637

**Published:** 2026-07-31

**Authors:** D. Andreou, N. E. Steen, A. M. Myhre, O. A. Andreassen, R. H. Yolken, I. Agartz

**Affiliations:** 1 Division of Mental Health and Substance Abuse, Diakonhjemmet Hospital; 2 Section for Clinical Psychosis Research, Division of Mental Health and Addiction, Oslo University Hospital; 3 Centre for Precision Psychiatry, Division of Mental Health and Addiction, Oslo University Hospital and University of Oslo; 4 Departement of Research and innovation, Division of Mental Health and Addiction, Oslo University Hospital, Oslo, Norway; 5 Stanley Division of Developmental Neurovirology, Department of Pediatrics, Johns Hopkins University School of Medicine, Baltimore, MD, United States

## Abstract

**Introduction:**

Herpes simplex virus 1 (HSV1) is a neurotropic virus capable of establishing latent, persistent infection after initial exposure. HSV1 has been associated with cognitive impairments in adult patients with psychosis; however, this association has not been examined in adolescent psychosis.

**Objectives:**

We assessed HSV1 status (seropositive vs. seronegative) and cognition in adolescent patients with psychosis (early-onset psychosis; EOP) and healthy controls. We hypothesized that seropositive participants would show poorer cognitive performance than seronegative participants, and that the association would be stronger in or restricted to patients.

**Methods:**

We included a total of 109 participants: 37 with EOP (mean age=16 years, 73% females, 27% seropositive), and 72 healthy controls (mean age=16, 53% females, 19% seropositive). In the EOP-group, 34 had non-affective psychosis (19 schizophrenia spectrum disorders and 15 other non-affective psychoses). Three participants had an affective psychosis (one bipolar I disorder and two major depressive disorder). We utilized the Measurement and Treatment Research to Improve Cognition in Schizophrenia (MATRICS) Consensus Cognitive Battery. We evaluated circulatory immunoglobulin G concentrations expressed as a dichotomous measure. In our main analyses, we applied sex- and age-adjusted models to test associations between HSV1 status and MATRICS cognitive domain scores.

**Results:**

In the whole group of patients and healthy controls, HSV1 seropositivity was associated with impaired speed of processing (p=0.012), working memory (p=0.006), verbal learning (p=0.002), visual learning (p=0.049) and reasoning/problem solving (p=0.005), with medium to large effect sizes (partial eta-squared=0.06-0.09). For speed of processing, working memory, verbal learning and reasoning/problem solving, the associations were driven by the patient group, with large effect sizes (partial eta-squared=0.12-0.23).

**Conclusions:**

HSV1 exposure in adolescents was linked to reduced cognitive performance, an effect that was more pronounced among those with psychosis.

**Disclosure of Interest:**

D. Andreou: None Declared, N. E. Steen: None Declared, A. Myhre: None Declared, O. Andreassen Consultant of: CorTechs and Precision Health, Speakers bureau of: Janssen, Lundbeck and Sunovion, R. Yolken: None Declared, I. Agartz Speakers bureau of: Lundbeck

